# Genome-Wide Association Study of Psoriasis, Psoriatic Arthritis, Anti–TNF-α Response, and Polygenic Risk Score in a Russian Cohort

**DOI:** 10.3390/ijms27104422

**Published:** 2026-05-15

**Authors:** Arfenya E. Karamova, Anastasiia A. Buianova, Anastasiia A. Vorontsova, Alexey A. Kubanov

**Affiliations:** 1 State Research Center of Dermatovenereology and Cosmetology, 3, bld. 6, Korolenko St., 107076 Moscow, Russia; vorontsova@cnikvi.ru (A.A.V.); alex@cnikvi.ru (A.A.K.); 2 Laboratory of Clinical Genomics and Bioinformatics, Veltischev Research and Clinical Institute for Pediatrics and Pediatric Surgery of the Pirogov Russian National Research Medical University, 2, bld. 1, Taldomskaya St., 125412 Moscow, Russia; anastasiiabuianova97@gmail.com

**Keywords:** biologic agents, TNF-α inhibitors, pharmacogenomics, psoriasis, psoriatic arthritis, polygenic risk scores, adalimumab, infliximab, etanercept

## Abstract

Psoriasis is an immune-mediated inflammatory disease with a genetic component, characterized by dysregulation of cytokine signaling and activation of T lymphocytes. This study investigated genetic variants associated with psoriasis, psoriatic arthritis (PsA), and response to tumor necrosis factor alpha (TNF-α) inhibitors (adalimumab, infliximab, and etanercept) in a Russian cohort. A genome-wide association study (GWAS) was conducted in 1026 psoriasis patients and 9212 controls using Infinium Global Screening Array-24 v3.0 microarrays. Exploratory analyses of treatment response (*n* = 48) and PsA (*n* = 96) were performed without covariate adjustment or explicit modeling of population structure. Polygenic risk scores (PRS) were derived from internally estimated effect sizes in a split-sample design. The GWAS replicated a robust association in the major histocompatibility complex (MHC) region (rs12189871 near *HLA-C*, *p* = 3.2 × 10^−50^, OR = 2.99 [2.59–3.45]). Additional loci included variants in *ZC3H8* and *PLCL2*. Nominal signals were observed for *IL18R1*/*IL18RAP* in treatment response (including rs17027071) and for *RCL1* and *FBLIM1* in PsA; these findings remain exploratory. PRS demonstrated moderate predictive performance (AUC = 0.6355) and should be interpreted with caution given the study design. Overall, the results highlight a strong MHC signal in psoriasis, while findings for PsA and treatment response remain hypothesis-generating and require independent validation.

## 1. Introduction

Psoriasis is an immune-mediated disease whose pathogenesis is initiated by activation of cutaneous dendritic cells followed by stimulation of T lymphocytes. A central role in the maintenance of chronic inflammation and keratinocyte hyperproliferation is played by a cytokine cascade involving interleukins (IL)-1/-1β/-6/-17/-23 and tumor necrosis factor-alpha (TNF-α) [1]. Tissue-resident memory T cells (TRM), which persist in the skin after lesion resolution and are capable of re-initiating inflammation upon exposure to various triggers, represent a critical pathogenic component. These triggers include exogenous factors (infections, skin trauma, lifestyle characteristics, medication use, humidity, cold climate, and air pollution) and endogenous factors (microbiota dysbiosis, stress, lipid metabolism disorders, and dysregulation of sex hormones) [2].

Psoriasis is characterized by marked clinical heterogeneity, with psoriasis vulgaris being the most prevalent form [3]. Nearly 75% of patients exhibit multimorbidity, including metabolic disorders (dyslipidemia, obesity, diabetes), gastrointestinal diseases (inflammatory bowel disease), and psychiatric conditions [4]. Comprehensive patient assessment requires not only evaluation of skin disease severity using standardized indices (PASI, DLQI), but also active screening for associated comorbidities, underscoring the need for a multidisciplinary management approach [3].

Psoriatic arthritis (PsA) is a progressive inflammatory joint disease that, in the absence of timely diagnosis and treatment, may lead to irreversible bone deformities and persistent functional impairment [5]. The risk of PsA is particularly high in patients with nail involvement and psoriatic lesions of the scalp, intergluteal, and perianal regions [6].

As of 2025, the global prevalence of psoriasis among adults is estimated at 4.4%, with the highest prevalence reported in East Asia (5.7%) and the lowest in Africa (1.7%). In Europe, the prevalence is approximately 4.6% [7]. According to the Psoriasis Patient Registry of the Russian Society of Dermatovenerologists and Cosmetologists (as of 3 June 2021), 3433 patients were registered (including 165 individuals under 17 years of age), of whom 28.9% (*n* = 868) also had PsA—exceeding the global average of 19.7% [8]. The majority of patients were male (60.5%), the mean age at disease onset was 31 years, and a positive family history of psoriasis was reported in nearly one quarter of patients (23.7%), whereas PsA among relatives was significantly less common (1.7%) [9]. The annual economic burden of psoriasis in Russia in 2018 (356,069 registered patients, corresponding to 242.4 cases per 100,000 population) amounted to 90.48 billion rubles, including 22.7 billion rubles in direct medical costs [10].

Concordance rates for psoriasis are significantly higher in monozygotic twins compared with dizygotic twins (0.33 vs. 0.17), with genetic factors accounting for approximately 68% of disease susceptibility variance [11]. Only four monogenic forms of psoriasis are currently registered in the OMIM database, associated with *HLA-C* (predominantly HLA-C*06:02 [12]), *CARD14*, *IL36RN*, and *AP1S3*. In recent years, large-scale genome-wide association studies (GWAS) have substantially advanced the understanding of psoriasis genetics. The largest meta-analysis to date, published in 2025, included 18 GWAS and identified 109 associated loci, including canonical immune response genes such as *IL23R*, *TNFAIP3*, *TRAF3IP2*, *TYK2*, and *RIGI* [13]. Notably, all included studies were conducted in European populations, limiting extrapolation to Russian cohorts, particularly for polygenic risk score (PRS) development. GWAS have also been conducted in other populations, such as South Asians [14]. Gene ontology and pathway enrichment analysis of 318 psoriasis-associated genes revealed predominant involvement of cytokine production, cytokine receptor activity, and the JAK–STAT signaling pathway [15]. The genetic basis of PsA remains less well characterized. Although some loci overlap with those associated with psoriasis, HLA-C*06:02 is not implicated in PsA susceptibility [16]. Instead, PsA is more strongly associated with polymorphisms in *IL23R* and *HLA-B* alleles (HLA-B*08, HLA-B*27, HLA-B*38, and HLA-B*39) [17]. A predictive algorithm based on 200 genetic markers has been developed to estimate PsA risk in patients with psoriasis, achieving an AUC of 0.82 [18].

Pharmacogenetics of response to biologic therapies, particularly TNF-α inhibitors, represents another important research direction [19]. Despite widespread clinical use of TNF-α inhibitors, few reproducible genetic predictors of treatment response in psoriasis have been identified. According to the ClinPGx database, only one variant–drug–phenotype association has achieved clinically actionable evidence level 2B: patients with the GG genotype at rs1800629 demonstrate the best response to etanercept, whereas AA carriers show reduced efficacy and AG carriers an intermediate response [20]. Five genes have been associated with adverse drug reactions (rs651630 *SLC12A8*, rs1135216 *TAP1*, rs3087243 *CTLA4*, rs11209026 *IL23R*, rs10782001 *FBXL19*), whereas 22 genes have been linked to treatment efficacy (*CDKAL1*, *CTNNA2*, *FCGR2A*, *FCGR3A*, *HLA-C*, *IL12B*, *IL17RA*, *IL1B*, *IL6*, *IVL*, *LY96*, *MAP3K1*, *NFKBIA*, *PGLYRP4*, *SLC9A8*, *SLCO1C1*, *TLR2*, *TLR9*, *TNF*, *TNFAIP3*, *TNFRSF1B*, *ZNF816*). The studied agents included adalimumab, infliximab, and etanercept.

PRS have demonstrated high predictive accuracy for cardiovascular diseases such as coronary artery disease and atrial fibrillation (AUC ≥ 0.76) [21]; however, their performance in psoriasis varies widely: AUC = 0.611 in a Taiwanese cohort [22], 0.76 in European patients [23], and 0.885 in a Chinese population when PRSs were combined with non-genetic covariates such as alcohol consumption, age, and gender [24]. In addition to yet-unidentified transethnic loci with stable effects and predominant genetic determination over environmental factors, a major limitation remains the lack of PRS specifically adapted to Eastern European and Russian populations.

## 2. Results

### 2.1. Exploratory Pharmacogenetic Analysis of TNF-α Inhibitor Response

**Figure 1:**
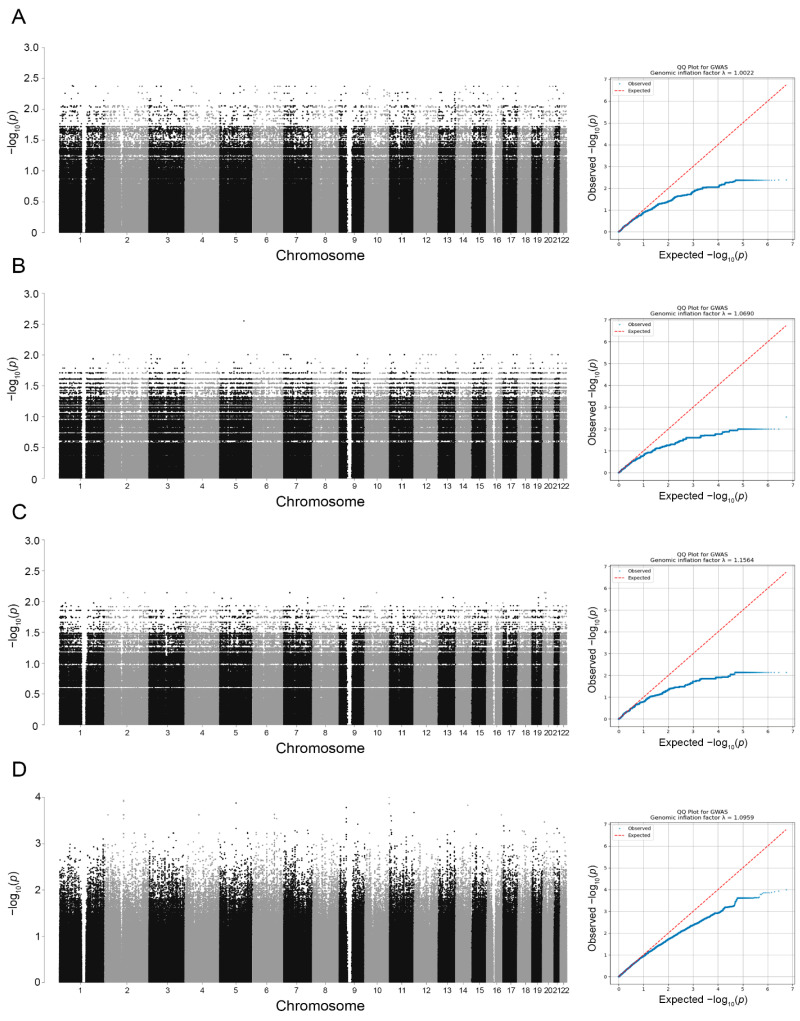
Quantile–quantile (Q–Q) plots and Manhattan plots of genetic markers associated with achievement of PASI 75 at week 16 in the study cohort. The figure presents *p*-values for associations between genetic variants and therapeutic response to adalimumab (**A**), infliximab (**B**), etanercept (**C**), and at least one TNF-α inhibitor (**D**) in patients with psoriasis. The Y-axis represents −log_10_(*p*) values obtained from additive logistic regression models, while the X-axis indicates chromosomal positions of genetic variants (hg19 assembly). No variant reached genome-wide significance (*p* < 5 × 10^−8^). PASI—Psoriasis Area and Severity Index.

GWAS was performed in 48 patients with known therapeutic response to biologic agents using additive logistic regression models. Manhattan and quantile–quantile (Q–Q) plots were generated for patients achieving Psoriasis Area and Severity Index (PASI) 75 at week 16 of therapy with adalimumab (Figure 1A), infliximab (Figure 1B), and etanercept (Figure 1C). In addition, logistic regression results for the combined TNF-α inhibitor cohort are presented (Figure 1D). The pooled analysis yielded the most robust association signals compared with individual drug analyses and was therefore prioritized for subsequent interpretation.

The 12 variants with the lowest *p*-values are presented in Table 1. Of these, 11 variants were associated with a poorer response to TNF-α inhibitor therapy, and one variant (rs12881315-T) was associated with achievement of PASI 75 at week 16. As all variants had adjusted *p*-values (*p*_adj_) > 0.05, the observed associations should be considered exploratory. Therefore, Table 1 reports unadjusted *p*-values. This limitation is attributable to the small sample size and the large number of tested variants.

Among the identified variants, only rs56044378–*IL18RAP*(NM_001393487.1):c.1210+1700G>A (located in intron 8) and rs17027071–*IL18R1*(NM_003855.5):c.1271–317C>T (located in intron 10) were situated within protein-coding genes. Variants rs3124044 and rs2993202 were located in the non-coding exon 2 of gene *ENSG00000284612*. Based on data from the CSP FMBA database [25], as well as allele A1 frequencies, variants rs9419590, rs7898015, rs4072670, and rs4072672 are likely to be in linkage disequilibrium (LD) in Russian genomes, representing a single haplotype. Another probable haplotype comprises variants rs3124044 and rs2993202.

**Figure 2:**
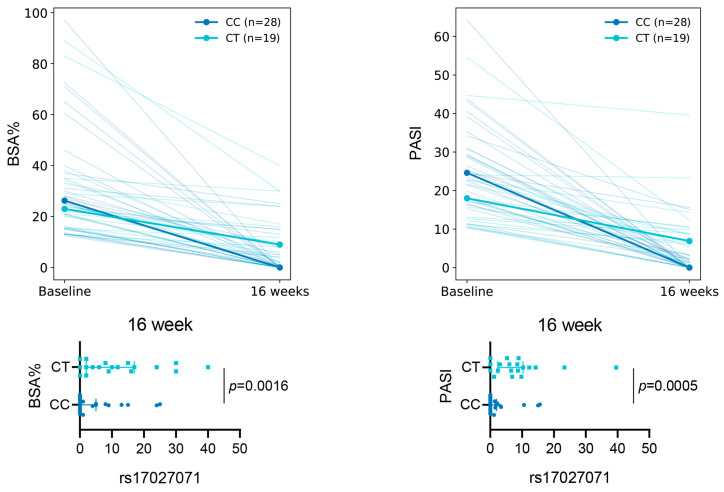
Dynamics of clinical parameters in patients with different rs17027071 genotypes. The plots illustrate changes in BSA (%) and PASI from baseline (week 0) to week 16 of therapy for patients with CC (dark blue) and CT (light blue) genotypes (one patient with the TT genotype was excluded from group comparisons due to insufficient sample size). Each line represents individual patient data. Comparisons between genotypes were performed using the Mann–Whitney U test. BSA—body surface area; PASI—Psoriasis Area and Severity Index.

Prior to therapy initiation, clinical indices BSA (body surface area) and PASI did not differ between patients carrying rs17027071 genotypes CC and CT (BSA *p* = 0.672, PASI *p* = 0.098). However, at week 16 of therapy, patients with the CC genotype demonstrated a significantly better therapeutic response: median BSA values were 0 [0–5] versus 9 [2–17], respectively (*p* = 1.64 × 10^−3^), and median PASI values were 0 [0–1.975] versus 6.9 [2.4–10.3] (*p* = 4.50 × 10^−4^) (Figure 2).

To evaluate the functional relevance of the identified genes *IL18R1* and *IL18RAP*, pathway enrichment analysis was performed using the Reactome database [26]. The most statistically significant pathway was “Interleukin-18 signaling” (R-HSA-9012546), with a false discovery rate (FDR) of 3.43 × 10^−6^, encompassing seven additional genes. Two out of four molecular reactions associated with this pathway involved the studied genes. The second-most significant pathway was “Interleukin-1 family signaling” (R-HSA-446652), with an FDR of 4.27 × 10^−4^, involving 142 proteins, of which two were identified, and participation in 6 of 92 reactions. The broader “Interleukin signaling” pathway (R-HSA-449147), integrating multiple interleukin-dependent cascades, was also significantly enriched (FDR = 0.00296). These findings indicate that *IL18R1* and *IL18RAP* are involved in a wider network of inflammatory and immune responses. More general pathways, such as “Cytokine signaling in the immune system” (R-HSA-1280215) and “Immune system” (R-HSA-168256), also showed significant enrichment (FDR < 0.05), despite involving a large number of molecules.

**Figure 3:**
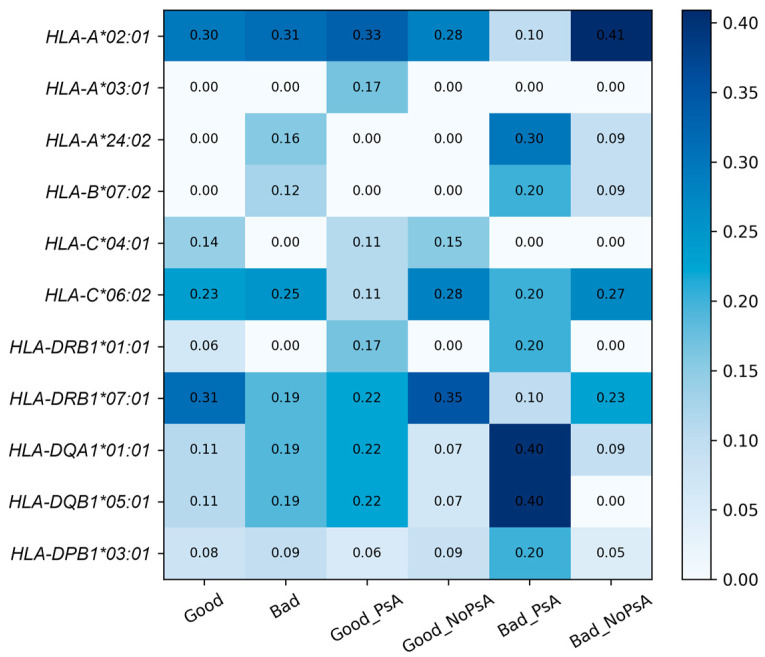
The heatmap shows the distribution of selected *HLA* alleles across clinical subgroups (good response, bad response, PsA status strata) (*n* = 48). Cell values represent allele frequencies within each subgroup, calculated as the proportion of allele occurrences relative to the total number of alleles observed in the corresponding group. PsA—psoriatic arthritis.

All reported *p*-values are nominal and were not adjusted for multiple testing and should therefore be interpreted as exploratory. In the good responder group, the *HLA*-A*03:01 allele was observed exclusively in patients with PsA compared to non-PsA patients (16.7% vs. 0%, *p* = 0.0196; nominal) (Figure 3). In the poor responder group, HLA-DQA1*01:01 and HLA-DQB1*05:01 were more frequently observed among PsA patients compared to non-PsA patients (40.0% vs. 9.1%, *p* = 0.0599; nominal), suggesting a possible trend toward enrichment of class II alleles in non-responders with joint involvement. Across the overall cohort, HLA-DRB1*01:01 was significantly more frequent in patients with PsA compared to those without PsA (17.9% vs. 1.5%; OR = 14.56, *p* = 0.0076; nominal). No association was observed between HLA-C*06:02 and response to adalimumab (62.5% vs. 45.5%, *p* = 0.395) or PsA status (14.3% vs. 27.9%, *p* = 0.194). Genotype distributions were consistent with Hardy–Weinberg equilibrium (HWE) across all loci and subgroups, with the exception of *HLA-DPB1* in the non-responder group (*p* = 0.03), which likely reflects sampling variability given the small subgroup size rather than true deviation.

### 2.2. Genetic Architecture of Psoriasis in the Russian Cohort

**Figure 4:**
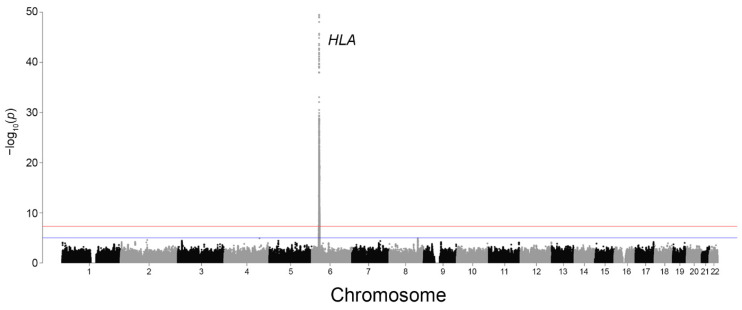
Manhattan plot of genetic markers associated with psoriasis (1026 patients and 9212 population controls). The blue horizontal line indicates the suggestive significance threshold (*p* = 1 × 10^−5^), and the red horizontal line indicates the genome-wide significance threshold (*p* = 5 × 10^−8^).

GWAS conducted in 1026 patients with psoriasis and 9212 population controls identified the strongest association signals on chromosome 6, with the most significant variant being rs12189871 (*p* = 3.2 × 10^−50^, OR = 2.99 [95% CI: 2.59–3.45], T allele frequency = 13.08%). In total, 3520 variants reached genome-wide significance (*p* < 5 × 10^−8^, the conventional threshold for GWAS; Figure 4). This large number of significant variants reflects extensive LD and the high genetic variability of chromosome 6.

A total of 732 variants representing 127 unique genes located on all chromosomes except chromosome 6 passed the statistical significance threshold (FDR < 0.01). The majority of variants were intronic (50%) or located in non-coding transcripts (23%). Additional variants were found downstream (9%) and upstream (8%) of annotated genes. Less frequent were variants associated with nonsense-mediated decay (NMD) transcripts (4%), intergenic variants (3%), and exonic variants of non-coding transcripts (1%). Approximately 1% of variants were located in regulatory regions.

Table 2 presents the 12 variants with the lowest *p*-values among the 732 significant variants. All were risk alleles, and none were present in the CSP FMBA database [25]. Variants with identical allele frequencies and ORs (95% CIs) were grouped into haplotypes. The variant within *ZC3H8* exhibited the lowest *p*-value among all variants located in protein-coding genes with established function (*p*_adj_ = 6.75 × 10^−4^). The most significant protective allele was chr3:17003915C>CT, *PLCL2*(NM_001144382.2):c.328-5759_328-5758insT (allele frequency 0.331; OR 0.769 [0.679–0.871]; *p*_adj_ = 3.59 × 10^−5^).

To assess the functional impact of the identified genes, pathway enrichment analysis was performed using the Metascape database [27]. Analysis of the top 400 variants passing Bonferroni correction demonstrated a significant association with psoriasis according to the DisGeNET database. Notably, only one *HLA* gene (*HLA-C*) was present among these variants. One of the enriched terms was “psoriatic arthritis” (C0003872), involving eight genes (*POU5F1*, *SKIC2*, *TNXB*, *HCP5*, *CCHCR1*, *PSORS1C1*, *MUC22*, and *LINC02571*), accounting for 33% of all genes implicated in disease pathogenesis (log_10_(*p*) = −8.90, log_10_(*p*) = −5.30). An association with psoriasis vulgaris (C0263361) was also identified, involving five genes (*CDSN*, *TCF19*, *CCHCR1*, *MUC22*, and *LINC02571*; 21%), log_10_(*p*) = −5.80, log_10_(*q*) = −2.60. Three genes (*CCHCR1*, *MUC22*, and *LINC02571*) were common to both phenotypes, suggesting a central role in the pathogenesis of the psoriatic disease spectrum.

In the conducted GWAS, several variants were localized within the *POU5F1* gene, including c.-184+6917C>A, c.-184+4393C>G, c.-184+3671A>G, c.-184+3525C>T, c.-184+3116C>A, c.-184+1426G>T, c.-183-305C>T (ENST00000441888.7), and ENST00000259915.13:c.757_758del (p.Pro253GlufsTer27). Downstream of *POU5F1*, the variants c.*3372C>T and c.*3024T>C (ENST00000441888.7) were identified. Four variants downstream of *SKIC2*—c.*4956G>A, c.*4613T>A, c.*2655C>G, and c.2387_2388insG (ENST00000375394.7)—were also detected, which may potentially influence regulatory regions of the genome. Within the *TNXB* gene, variants c.6544+896A>G, c.6842-621_6842-620del, c.9604G>A (p.Asp3202Asn), and c.9115+569_9115+580del (ENST00000644971.2) were mapped. In *MUC22*, variants c.71-5692C>T, c.71-5220C>A, and c.5056-809T>G (ENST00000561890.1) were identified. Additionally, six intragenic variants were found in *LINC02571*: n.171+653C>G, n.171+2006A>T, n.171+1634A>T, n.171+1590A>C, n.171+829G>T, and n.172-2548T>A (ENST00000539514.1). Nine variants were located near *HCP5*: n.5_8del, n.*51G>T (ENST00000414046.3), n.*90T>C, n.*242C>A, n.-252A>T, n.-427A>T, n.-856T>A, n.-1178A>C, and n.-1255T>A (ENST00000541196.3), alongside eleven intragenic variants: n.97T>G, n.198-90C>T, n.198-27A>C (ENST00000541196.3), n.3276C>G, n.3600A>G, n.4761T>C, n.4839G>A, n.4885C>G, n.5968C>T, n.6231G>C, and n.8297G>A (ENST00000414046.3). Only one variant was detected within *CDSN* (ENST00000376288.3:c.979G>T, p.Glu327Ter), with the remainder located upstream or downstream. Furthermore, the GWAS revealed one intronic variant in ENST00000376257.8:c.239-218G>A, one synonymous variant (c.369A>G), and two 3′-UTR variants (c.*444G>T and c.*921G>C).

Variants outside chromosome 6 that did not surpass the Bonferroni-corrected significance threshold but were significant by FDR (<0.01) were analyzed separately. The most statistically significant pathway enriched among the studied genes was the “complement receptor-mediated signaling pathway” (GO:0002430), comprising three genes (2.65%), with log_10_(*p*) = −4.40 and log_10_(*p*) = −0.06. Other enriched pathways included: “circadian behavior” (GO:0048512), 3 genes (2.65%), log_10_(*p*) = −3.96, log_10_(*q*) = −0.03; “apoptosis” (hsa04210), 5 genes (4.42%), log_10_(*p*) = −3.77, log_10_(*q*) = −0.03; “effects of omega-3 polyunsaturated fatty acids on Huntington’s disease signaling pathways” (WP5470), 4 genes (3.54%), log_10_(*p*) = −2.96, log_10_(*q*)~0; “calcium regulation in cardiac myocytes” (WP536), 4 genes (3.54%), log_10_(*p*) = −2.59, log_10_(*q*)~0; “ALK signaling pathway in cancer” (R-HSA-9700206), 3 genes (2.65%), log_10_(*p*) = −2.33, log_10_(*q*)~0; “intracellular protein transport” (GO:0006886), 7 genes (6.19%), log_10_(*p*) = −2.21, log_10_(*q*)~0; “microtubule cytoskeleton organization” (GO:0000226), 7 genes (6.19%), log_10_(*p*) = −2.17, log_10_(*q*)~0; and “protein homo-oligomerization” (GO:0051260), 4 genes (3.54%), log_10_(*p*) = −2.11, log_10_(*q*)~0. These enriched clusters reflect the involvement of the studied genes in immune activation, apoptosis, circadian regulation, oncogenic signaling, fatty acid metabolism, intracellular transport, and cytoskeletal architecture.

### 2.3. Construction of PRS for Psoriasis in the Russian Cohort

**Figure 5:**
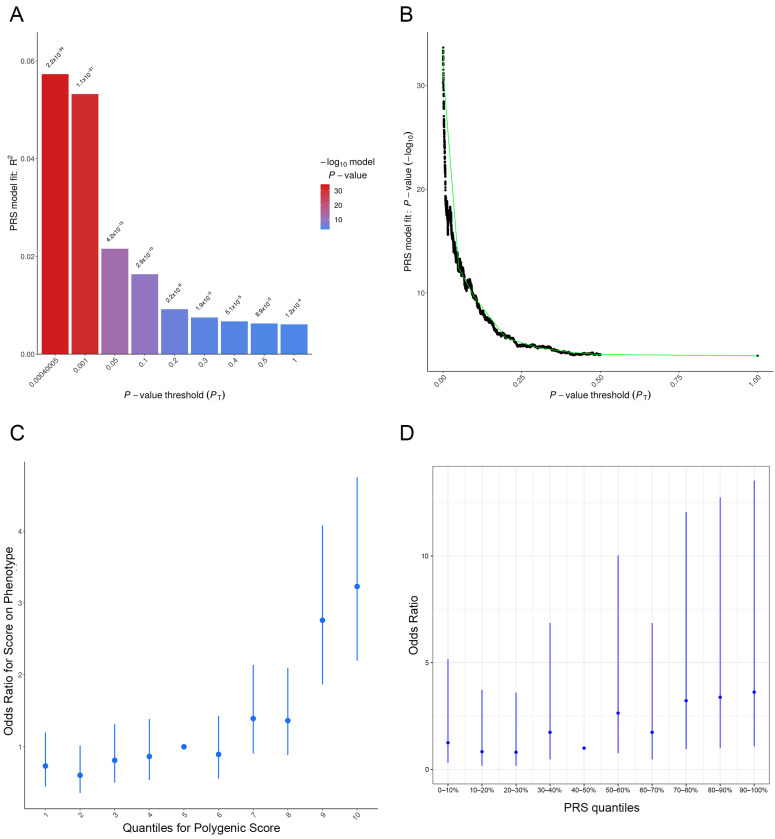
PRS model for psoriasis in the training cohort (136 variants) (**A**–**C**): (**A**) The X-axis shows *p*-value thresholds (P_t_) for SNP and indel inclusion in PRS calculation, while the Y-axis represents the proportion of phenotypic variance explained (R^2^). Bar color corresponds to −log_10_(*p*) of the model association, indicating statistical significance. (**B**) Relationship between explained variance (R^2^) and selected *p*-value thresholds. The green line connects key thresholds. (**C**) Stratified polygenic risk analysis. The X-axis represents PRS deciles (1–10), and the Y-axis shows odds ratios relative to the reference group (5th decile). Vertical lines denote 95% confidence intervals. (**D**) Model validation in a cohort of 96 patients with moderate-to-severe psoriasis and 239 controls. The X-axis represents PRS quantiles; the Y-axis shows odds ratios. A dose-dependent relationship between PRS magnitude and psoriasis risk is observed, with the most pronounced increase in the top 20% of the PRS distribution. Wide confidence intervals reflect the limited sample size in the validation cohort. PRS—polygenic risk score; SNP—single-nucleotide polymorphism.

A PRS model for psoriasis was developed using 136 genetic variants, yielding an AUC of 0.6522 in the training cohort (Figure 5A–C). Analysis of phenotype dependence on PRS demonstrated a gradual increase in OR across genetic risk score quantiles (Figure 5C). Compared with the reference group (5th decile, corresponding to average PRS values), individuals in higher quantile groups (starting from the 7th decile) showed increased relative risk, with the highest estimates observed in the upper deciles.

In the validation cohort, the PRS achieved an AUC of 0.6355 (95% CI: 0.5702–0.7009, DeLong method; *p* = 4.78 × 10^−5^), indicating moderate discriminative performance. Supplementary Table S1 lists the variants included in the PRS construction, along with their GWAS *p*-values from the additive model. Note that the *p*-values for the PRS itself, reported in the main text, differ from these individual variant *p*-values because the PRS represents a weighted aggregate of all variants rather than a single-variant association. Stratified PRS analysis (Figure 5D) showed that in the lower quantiles (0–40%), OR values were low and did not exceed 2, with wide confidence intervals crossing 1, indicating no clear association between PRS and psoriasis risk in these strata. The 40–50% range corresponded to the lowest OR and was used as the reference (baseline risk) group. Starting from the 60–70% quantile, OR values increased, with the most pronounced effect observed in the upper deciles. In the 90–100% quantile, OR exceeded 10, suggesting higher relative risk among individuals with the highest PRS values; however, confidence intervals remained wide, reflecting limited sample sizes within strata and uncertainty of estimates. Overall, the observed gradient reflects relative enrichment of cases at higher PRS levels rather than a clinically actionable predictive model.

**Figure A1:**
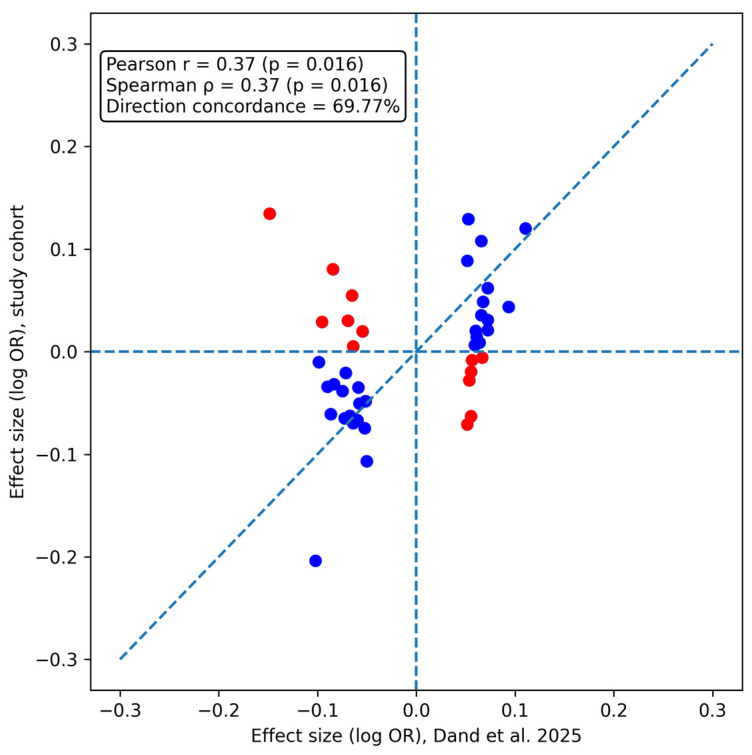
Comparison of effect sizes between the study cohort and published GWAS data. Each point represents a variant present in both datasets after harmonization of effect alleles. The X-axis shows log-transformed odds ratios from the external GWAS (Dand et al., 2025 [13]), and the Y-axis shows corresponding estimates from the study cohort. A diagonal line indicates perfect concordance of effect sizes, and dashed lines represent the null effect (log OR = 0). Blue points indicate variants with concordant direction of effect between the two datasets, whereas red points indicate variants with discordant effect directions.

To assess the consistency of genetic associations with external evidence, we compared effect estimates between our cohort and a large-scale psoriasis GWAS (Dand et al., 2025 [13]). After harmonization of effect alleles, 43 overlapping variants were identified. A moderate positive correlation was observed between effect sizes in the two datasets (Pearson r = 0.37, *p* = 0.016; Spearman ρ = 0.37, *p* = 0.016) (Figure A1). In addition, 69.8% of variants demonstrated concordant effect directions, exceeding the expectation under the null hypothesis of random agreement (50%). These results indicate consistency of genetic effect estimates with previously reported psoriasis GWAS findings.

### 2.4. Exploratory GWAS of PsA

The GWAS aimed at identifying genetic determinants of PsA did not reveal any variants reaching genome-wide significance (*p* < 5 × 10^−8^). The 15 variants with the lowest *p*-values are presented in Table 3; among them, 11 represent risk alleles and 4 are protective.

The most significant associations were observed across 141 genes and did not reach genome-wide significance after multiple testing correction. Among these, the strongest signals were enriched in scoliosis-related phenotypes, including isolated type III scoliosis (C1837461) and adolescent idiopathic scoliosis (C0410702), involving 12 genes (9.4%) with log_10_(*p*)/log_10_(*q*) values of −5.2/−1.5 and −4.7/−1.1, respectively (*OPCML*, *PTPRD*, *MYOM1*, *ZNF423*, *ASTN2*, *FRMD4A*, *SORCS2*, *FAM184A*, *SNX29*, *TMEM132C*, *TSHZ2*, *PRICKLE2*).

Phenotype enrichment analysis revealed nominal associations with a broad spectrum of traits, including smoking behavior, cardiometabolic measures (e.g., blood pressure [C0428883] and waist-to-hip ratio [C0205682]), respiratory function parameters (e.g., forced expiratory volume and pulmonary function tests), and neuropsychiatric phenotypes (narcolepsy, neurodevelopmental disorders, and depressive bipolar disorder).

Given the absence of genome-wide significant signals in the PsA cohort, these results should be interpreted cautiously. Rather than indicating PsA-specific genetic determinants of comorbid conditions, the observed enrichment likely reflects shared polygenic architecture across complex inflammatory, cardiometabolic, and neuropsychiatric traits.

## 3. Discussion

In this study, we investigated the genetic architecture of psoriasis, PsA, and response to TNF-α inhibitors in a Russian cohort using a multi-layered genomic approach. The most robust findings were obtained from the psoriasis susceptibility GWAS, while the pharmacogenetic and PsA analyses should be interpreted as exploratory due to limited statistical power.

The most significant variant was rs12189871, located near the *HLA-C* gene. It has previously been described as one of the strongest genetic risk factors for cutaneous psoriasis and showed a more pronounced association with skin manifestations of the disease compared with PsA [28]. Furthermore, the clinical relevance of rs12189871 has been supported by pharmacogenomic studies demonstrating its association with response to targeted therapy. Specifically, this variant, together with other *HLA*-region single-nucleotide polymorphisms (SNPs) and the classical *HLA-Cw6* allele, has been linked to a high clinical efficacy of the IL-17A inhibitor secukinumab [29]. Interestingly, the second-most significant variant in our cohort, rs12199223 (*p* = 3.88 × 10^−50^, OR = 2.99 [2.59–3.45], T allele frequency 13.09%), is consistent with findings from an Egyptian GWAS, which reported genome-wide significant associations at rs12199223 (*p*_comb = 6.57 × 10^−18^) and nearby rs1265181 (*p*_comb = 1.03 × 10^−10^) in the major histocompatibility complex (MHC) region [30]. This concordance highlights the conserved role of the MHC locus across diverse populations while also suggesting potential interethnic differences in allele frequency and effect size.

In the psoriasis susceptibility GWAS, expected associations within the *HLA* region were replicated, along with significant signals outside chromosome 6. Among these, a variant in *ZC3H8*, which encodes a zinc-finger protein acting as a negative regulator of immune responses, was identified. ZC3H8 expression is markedly increased in psoriasis, suggesting a compensatory attempt to suppress inflammation [31]. A protective variant in *PLCL2* highlights the potential involvement of B-cell-mediated pathways in psoriasis pathogenesis. *PLCL2* encodes a phospholipase C–like protein that negatively regulates B-cell receptor signaling and contributes to B-1a B-cell differentiation. Although psoriasis is traditionally considered a T-cell-mediated disease, involvement of PLCL2 suggests a broader role for adaptive immunity. In a large GWAS meta-analysis (>15,000 psoriasis cases and >27,000 controls), *PLCL2* (locus 3p24.3) was significantly associated with psoriasis (*p* = 3.7 × 10^−8^), despite the absence of pathogenic variants within its coding region. This locus has also been implicated in primary biliary cholangitis, another autoimmune disorder [32].

SNPs located up to 26 kb upstream of *HLA-C*, within regions potentially transcribing long non-coding RNAs (lncRNAs), including *LINC02571*, may influence HLA-C class I expression in immune and skin-resident cells. This, in turn, could affect the expansion of CD8^+^ T cells producing IFN-γ and IL-17.

Other genome-wide significant variants were located in *POU5F1* [33], *SKIC2* [34], *TNXB* [35], *MUC22* [36], *HCP5* [37,38], *CDSN* [39], and *TCF19* [40].

*HCP5* (HLA Complex P5) is a long non-coding RNA gene derived from an endogenous retroviral element and expressed predominantly in lymphocytes. GWAS in U.S. and U.K. populations previously identified rs2395029-C as the second-most significant risk factor for psoriasis (*p* = 2.13 × 10^−26^) [37]. Its role has also been confirmed in Indian and Chinese populations, with stronger associations observed for type I and moderate-to-severe psoriasis [38]. *CDSN* gene encodes corneodesmosin, a protein localized to corneodesmosomes in keratinizing epithelia. *CDSN* is highly polymorphic, and its variants are associated with skin disorders including psoriasis, hypotrichosis, and peeling skin syndrome. The gene is located within the MHC class I region [39].

The observed signals involving *IL18R1* and *IL18RAP* should be interpreted within the context of an exploratory pharmacogenetic analysis conducted in a limited sample size (*n* = 48), in which none of the associations reached genome-wide significance after correction for multiple testing. Accordingly, these findings do not provide evidence of causality and should not be interpreted as validated genetic determinants of therapeutic response.

Despite these limitations, IL18R1 and IL18RAP are biologically plausible candidates based on prior literature, given their established role as components of the IL-18 receptor complex. IL-18 is a proinflammatory cytokine expressed in the skin by keratinocytes and Langerhans cells. In psoriasis, IL-18 levels are markedly elevated, especially in the suprabasal layers of the epidermis, consistent with a role in cutaneous inflammation. IL-18 signals through the IL18R1/IL18RAP receptor complex, which is required for activation of downstream pathways, including NF-κB and JNK. Expression of IL-18Rα on keratinocytes is upregulated by IFN-γ and TNF-α, rendering these cells more responsive to IL-18. Upon IL-18 stimulation, keratinocytes increase CXCL10 production, promoting Th1 cell recruitment, and upregulate MHC class II expression, enabling participation in T-cell activation. Enhanced IL-18 sensitivity in psoriatic skin may therefore contribute to the maintenance of chronic inflammation [41]. Plasma IL-18 levels have been shown to correlate with psoriasis severity. Furthermore, in the presence of IL-4 and absence of IL-12, IL-18 may promote Th2 differentiation [42].

Although the PsA GWAS did not achieve genome-wide significance due to limited statistical power, the identified associations and haplotypes—particularly within the *RCL1*, *FBLIM1*, *TMEM132C*, *ENSG00000307024*, and non-coding RNA loci *LINC02363/LINC02362*—warrant further validation. At the nominal level, enrichment for smoking-related, metabolic, and neuroimmune phenotypes was observed, aligning with the known clinical associations of PsA, but these findings require replication. Elevated body mass index (BMI) is known to correlate with psoriasis risk [43] and, specifically, with PsA [44].

The observed *HLA* patterns may suggest a differential contribution of class I and class II alleles to psoriasis phenotypes and treatment response. Nominal associations involving class II alleles, particularly HLA-DRB1*01:01, HLA-DQA1*01:01, and HLA-DQB1*05:01, point toward a potential role of CD4^+^ T-cell-mediated immune responses in the development of psoriatic arthritis. This is further supported by previous studies identifying HLA-DRB1*01:01 as a susceptibility allele for PsA in independent populations [45]. In contrast, HLA-C*06:02, the principal genetic determinant of cutaneous psoriasis, did not demonstrate an association with either treatment response or PsA in our cohort. This is in line with prior reports showing that HLA-C*06:02 is primarily linked to skin-limited disease rather than joint involvement [16]. Moreover, the lack of association between HLA-C*06:02 and response to adalimumab in our cohort concurs with some but not all previous reports [46], possibly due to limited statistical power. Although none of the *HLA* associations remained significant after correction for multiple testing, the concentration of nominal signals within the *HLA* region reflects the central role of antigen presentation in psoriasis pathogenesis.

The PRS model demonstrated moderate predictive performance and a pronounced increase in risk in the upper PRS deciles. However, given the limitations in PRS construction (internal weights, likely overestimation of AUC), independent external validation is required before any clinical application can be considered.

## 4. Materials and Methods

### 4.1. Patients

The study included 96 patients with psoriasis vulgaris of moderate-to-severe severity, including 31.2% (*n* = 30) with concomitant PsA. Patient age ranged from 18 to 76 years (median = 43.94; interquartile range [IQR]: 35–55), and disease duration ranged from 2 to 55 years (median = 17; IQR: 10–28). The cohort consisted of 76 men and 20 women.

Disease severity was assessed using standardized clinical indices: BSA and PASI. The median affected BSA was 28% (IQR: 20–45; data missing for one patient), and the median PASI score was 21.45 (IQR: 16.43–29.08). Scalp involvement was observed in 90.63% of patients, and psoriatic nail dystrophy in 50%.

A positive family history of psoriasis was reported by 28.13% (*n* = 27) of patients. Among factors and comorbidities aggravating disease course, 22.92% (*n* = 22) were smokers, one patient reported alcohol abuse, 26.04% (*n* = 25) had obesity, and 35.42% (*n* = 34) were overweight. The mean BMI was 27.1 ± 5.32 kg/m^2^. Hypertension was present in 26.04% (*n* = 25) of patients; high cardiovascular risk was identified in 13.54% (*n* = 13), and moderate risk in 20.83% (*n* = 20). Multiple lines of biologic therapy had been administered to 15.63% (*n* = 15) of patients.

To identify genetic markers of biologic therapy efficacy, 48 patients with differing therapeutic responses to TNF-α inhibitors (adalimumab, infliximab, etanercept) were selected from the total cohort of 96 patients for comparative genotyping analysis.

The subgroup of 48 patients included 40 men (83.33%) and 8 women (16.67%), with a mean age of 46.94 ± 13.93 years. PsA was diagnosed in 14 patients (29.17%). Median disease duration was 17.5 years (IQR: 11.5–29.75). Baseline disease severity in all patients met criteria for moderate (10 ≤ PASI < 20) or severe (PASI ≥ 20) psoriasis. Median BSA was 25% (IQR: 15.5–37; data missing for one patient), and median PASI was 22.8 (IQR: 15.95–30.45).

Scalp involvement was present in 43 patients (89.58%), nail dystrophy in 20 (41.67%), and inverse psoriasis in 7 (14.58%). A family history of psoriasis was reported by 15 patients (31.25%). Smoking was reported by 31.25% of patients, and one patient reported alcohol abuse. The mean BMI was 27.31 ± 4.8 kg/m^2^: normal weight in 15 patients (31.25%), overweight in 20 (41.67%), class I obesity in 6 (12.5%), class II obesity in 5 (10.42%), and underweight in 2 (4.17%). Hypertension was present in 16 patients (33.33%), high cardiovascular risk in 9 (18.75%), and moderate risk in 10 (20.83%). Prior therapy with interleukin inhibitors had been administered to 9 patients (18.75%).

Clinical efficacy of therapy was assessed at week 16 based on achievement of PASI 50/75/90/100 responses. A good response was defined as PASI ≥ 75, whereas an unsatisfactory response was defined as PASI ≤ 50.

**Figure 6:**
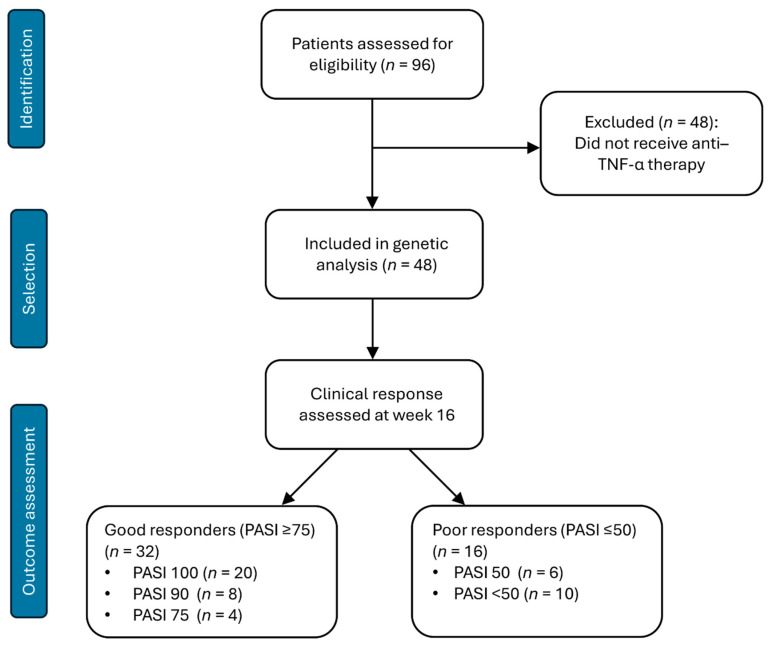
Flow diagram of patient selection and clinical response classification. A total of 96 patients with moderate-to-severe plaque psoriasis were initially included. Forty-eight patients receiving anti-TNF-α therapy with available clinical response data were selected for genetic analysis. Clinical efficacy was evaluated at week 16 using PASI improvement thresholds, and patients were classified as good responders (PASI ≥ 75) or poor responders (PASI ≤ 50). PASI—Psoriasis Area and Severity Index.

Patient distribution according to treatment efficacy is presented in Figure 6. By week 16 of therapy, 32 patients (66.67%) achieved target PASI 75/90/100 responses.

Clinical and anamnestic characteristics of patients with good and insufficient response to therapy are presented in Table 4.

### 4.2. DNA Genotyping

DNA concentration was measured using a Fluoroskan fluorescent microplate reader (Thermo Fisher Scientific, Waltham, MA, USA). DNA quality and integrity were assessed by agarose gel electrophoresis with band visualization. Sample preparation and microarray scanning were performed at Genotek LLC (Moscow, Russia) using the iScan System (Illumina, San Diego, CA, USA) in accordance with the Infinium HTS Assay Guide (Illumina).

Genotyping was carried out using Infinium Global Screening Array-24 v3.0 microarrays (Illumina), comprising 654,027 SNPs and indel markers, ensuring high genomic coverage and reproducibility. An AutosomalCallRateThreshold of 0.01 was applied, followed by internal genotype quality control filtering. Genotype imputation was performed using Beagle v5.0 (University of Washington, Seattle, WA, USA) [47] with parameters Ne = 20,000 and k = 90. European populations from the 1000 Genomes Project Phase 3 served as the reference panel. Post-imputation filtering was based on an imputation quality threshold of info score > 0.5 and genotype posterior probability > 0.5. These relatively permissive thresholds were intentionally chosen to retain low-frequency and immunogenetically relevant variants, particularly within highly polymorphic regions such as the *HLA* locus, for downstream exploratory pharmacogenetic analyses. This strategy prioritized sensitivity to potentially biologically relevant signals over strict genotype certainty, while acknowledging an increased level of uncertainty in retained variant calls.

### 4.3. GWAS of Biologic Therapy Response

The study methodology included identification of genetic markers associated with response to biologic therapies through comparative analysis of genotyping data from patients with moderate-to-severe psoriasis stratified by treatment response (*n* = 48). GWAS was performed: (1) for the combined group of TNF-α inhibitors (adalimumab, infliximab, etanercept); and (2) separately for each TNF-α inhibitor.

GWAS was conducted using PLINK2 (v2.0.0-a.6.9) (Complete Genomics, Mountain View, CA, USA) [48]. Variants genotyped in less than 95% of samples or with genotyping quality below 90% were excluded. The maximum relatedness coefficient (PI_HAT) was 0.2746, indicating the absence of first- or second-degree relatives within the cohort. Minor allele count was 5, variants deviating from HWE (*p* < 0.00001) were excluded. Association testing was performed using logistic regression under an additive genetic model, without covariates (e.g., age, sex, or principal components). *p*-values were adjusted for multiple testing using the Bonferroni correction (*p*_adj_ < 0.05). A total of 5,539,731 variants were analyzed. Variant annotation was conducted using Variant Effect Predictor (VEP) v113 (European Bioinformatics Institute, Hinxton, UK) [49].

*HLA* genotyping was performed from genomic data using HLA-HD v1.7.0 (Kyoto University Graduate School of Medicine, Kyoto, Japan) [50]. Classical *HLA* loci (class I: *HLA-A*, *-B*, *-C*; class II: *HLA-DRB1*, *-DQA1*, -*DQB1*, *-DPB1*) were inferred at 2-field (four-digit) resolution based on the IMGT/HLA reference database. Allele and haplotype frequencies were calculated, and comparisons between groups were performed using Fisher’s exact test. To account for multiple testing, FDR correction using the Benjamini–Hochberg procedure was applied separately within each subgroup. Deviation from HWE was assessed using chi-square tests within each group.

### 4.4. GWAS of Psoriasis

The analysis included 1026 patients with psoriasis and 9212 population controls from the Genotek database. Single-variant association analysis was performed using logistic regression under an additive genetic model in PLINK2 (v2.0.0-a.6.9) (Complete Genomics) [48]. All study samples were of Russian ancestry and were assumed to be relatively homogeneous at the population level. Therefore, no principal component (PC)-based correction for population stratification was applied. *p*-values were adjusted for multiple testing using the Bonferroni correction (*p*_adj_ < 0.05). A total of 6,439,541 variants were tested. Variant annotation was performed using VEP v113 (European Bioinformatics Institute) [49]. In addition to Bonferroni-corrected primary analyses, an exploratory FDR (Benjamini–Hochberg *q* < 0.01) framework was applied to facilitate detection of associations outside the extended LD structure of chromosome 6, where Bonferroni correction was overly conservative due to strong signal clustering.

The Genotek cohort was randomly split into two equal non-overlapping subsets. The first subset was used exclusively for GWAS to derive effect size estimates (log-odds ratios), which were subsequently used as weights for PRS construction. The second subset was used solely for PRS parameter tuning (*p*-value threshold selection) using PRSice-2 v2.3.3 (Mount Sinai, New York, NY, USA) [51], without re-estimation of effect sizes. The PRS for each individual was calculated as the weighted sum of risk alleles:

(1) $$PRS\;=\;\sum ln\left(OR_{i}\right)\cdot x_{i}\;$$

where $ln(OR_{i}$) is the natural logarithm of the odds ratio for the effect allele of the *i*-th variant (derived from the GWAS on the first subset), and $x_{i}$ is the allele count (0, 1, or 2). PRSice-2 tested multiple *p*-value thresholds (*P*_T_) for variant inclusion, and the optimal model was selected based on the highest variance explained (R^2^) in the training subset.

The optimized PRS model was validated in an independent cohort of 96 clinically verified patients with moderate-to-severe psoriasis (from the National Medical Research Center of Dermatology and Cosmetology) and 239 population controls (from the Genotek database), which were not involved in either the GWAS or tuning steps. Model performance was assessed by the area under the receiver operating characteristic curve (AUC), with 95% confidence intervals calculated using the DeLong method. Statistical significance was determined using a one-sided test for AUC > 0.5. Given that effect sizes were derived from the same cohort, the reported AUC may be upwardly biased and should be interpreted as an exploratory internal validation.

### 4.5. GWAS of PsA in Patients with Moderate-to-Severe Psoriasis

Within the study, a comparative analysis of genetic profiles was performed among patients with psoriasis (*n* = 96, National Medical Research Center of Dermatology and Cosmetology cohort), stratified by the presence of concomitant PsA: patients with psoriasis without arthritis (*n* = 66) were compared directly to patients with clinically confirmed PsA (*n* = 30). Association testing was performed at the single-variant level using logistic regression under an additive genetic model in PLINK2 (v2.0.0-a.6.9) (Complete Genomics), without covariates [48]. *p*-values were adjusted for multiple testing using the Bonferroni correction (*p*_adj_ < 0.05). A total of 5,539,731 variants were analyzed. Variant annotation was performed using VEP v113 (European Bioinformatics Institute) [49].

### 4.6. Statistical Power Analysis

Formal power calculations were performed using the two-proportion test (pwr.2p2n.test in R v4.5.2) (R Core Team, Vienna, Austria) with Bonferroni correction for multiple testing (α = 0.05/number of tested variants). For the pharmacogenetic analysis (32 responders vs. 16 non-responders) and for the PsA analysis (30 cases vs. 66 controls), power was estimated assuming an additive genetic model, MAF = 0.2, and OR = 3 and 10.

## 5. Conclusions

### Limitation of Our Study

Despite the multi-layered genomic design and comprehensive analytical framework, several limitations should be considered when interpreting the findings of this study.

First, the pharmacogenetic analysis of response to TNF-α inhibitors was conducted in a relatively small cohort (*n* = 48), which substantially limited statistical power to detect variants with modest or small effect sizes. Formal power calculations showed that for OR = 3 and MAF = 0.2 the power was <0.001, and even for OR = 10 it remained only 0.014. Consequently, no associations reached genome-wide significance after correction for multiple testing, and all observed signals should be interpreted as exploratory and hypothesis-generating rather than confirmatory.

Second, the analysis of PsA was constrained by a limited number of cases (*n* = 30 within a cohort of 96 patients). Power calculations revealed <0.001 for OR = 3 (MAF = 0.2) and 0.208 for OR = 10. This precluded robust identification of PsA-specific genetic determinants and increases the likelihood of both false-positive and false-negative findings. Accordingly, the reported associations and pathway enrichments should be regarded as preliminary and require validation in larger, independent cohorts.

Third, the PRS was derived using effect size estimates obtained from the same cohort rather than from an independent large-scale GWAS. This does not follow the recommended practice of using external summary statistics, which is the gold standard for minimizing overfitting. Although a split-sample design was applied to partially mitigate this limitation, the absence of external weights likely leads to optimistic performance estimates. The reported AUC (0.6355) should therefore be interpreted with explicit recognition of this major limitation, and independent external validation in a separate Russian cohort is essential.

Fourth, the psoriasis GWAS was conducted without explicit adjustment for population stratification using PCA. Although the cohort is of Russian ancestry, well-documented genetic substructure within the population (including Slavic, Finno-Ugric, and Turkic components) may introduce residual confounding, particularly for associations outside the MHC region. This represents a recognized limitation of GWAS in ancestrally heterogeneous populations, and replication in independent cohorts with appropriate ancestry correction is essential.

Fifth, the genotype imputation strategy involved non-default Beagle v5.0 parameters (Ne = 20,000; k = 90) and relatively permissive post-imputation filtering thresholds (info score > 0.5; genotype posterior probability > 0.5). These choices were made to prioritize retention of low-frequency and immunologically relevant variation, particularly within the highly polymorphic *HLA* region. However, such settings may increase uncertainty in genotype likelihood estimation and introduce additional noise compared to more conservative GWAS filtering standards (e.g., info > 0.7–0.8). Although downstream analyses were focused on robust signals and replicated patterns, residual imputation uncertainty cannot be fully excluded and may increase uncertainty in effect estimates.

Finally, despite the application of rigorous multiple testing correction strategies, the high dimensionality of genomic data inherently increases the risk of residual false-positive associations, particularly in stratified and exploratory analyses. In addition, functional interpretations based on pathway enrichment analyses remain inferential and do not establish causality, thereby necessitating further experimental validation at the transcriptomic and proteomic levels.

## Figures and Tables

**Table 1:** List of 12 genetic markers with the lowest *p*-values associated with achievement of PASI 75 at week 16 of treatment with the combined TNF-α inhibitor group (adalimumab/infliximab/etanercept) using an additive logistic regression model. Number of patients: 48.

rsID	Gene	CHR	Genomic Position (hg19)	Effect Allele (A1)	Other Allele (A2)	Allele Frequency (A1)	Allele Frequency in CSP FMBA Database	OR (95% CI)	*p*
rs9419654	*ENSG00000303684/* *ENSG00000297817*	10	133,565,632	G	A	0.24	0.592	0.056 [0.013–0.239]	0.0001006
rs17027071	*IL18R1*	2	103,012,674	T	C	0.22	0.584	0.0572 [0.013–0.245]	0.0001167
rs56044378	*IL18RAP*	2	103,065,367	A	G	0.19	0.577	0.050 [0.011–0.231]	0.0001226
rs35213754	*ENSG00000255647/* *MBLAC2*	5	89,751,809	TA	T	0.19	0.738	0.013 [0.001–0.120]	0.0001333
rs9419590	*ENSG00000303684/* *ENSG00000297817*	10	133,565,224	G	A	0.26	0.592	0.062 [0.015–0.258]	0.0001375
rs7898015	*ENSG00000303684/* *ENSG00000297817*	10	133,567,096	C	T	0.26	0.572	0.062 [0.015–0.258]	0.0001375
rs4072670	*ENSG00000303684/* *ENSG00000297817*	10	133,568,101	A	G	0.26	0.591	0.062 [0.015–0.258]	0.0001375
rs4072672	*ENSG00000303684/* *ENSG00000297817*	10	133,568,224	A	G	0.26	0.591	0.062 [0.015–0.258]	0.0001375
rs12881315	*FLRT2/* *ENSG00000301142*	14	86,298,306	T	C	0.31	0.615	16.028 [3.822–67.206]	0.0001486
rs3124044	*ENSG00000284612*	9	38,660,694	G	A	0.31	0.576	0.062 [0.014–0.263]	0.0001666
rs2993202	*ENSG00000284612*	9	38,662,269	T	C	0.31	0.573	0.062 [0.014–0.263]	0.0001666
rs7129840	*ENSG00000297868/* *ENSG00000304250*	11	134,756,307	C	T	0.25	0.577	0.070 [0.017– 0.286]	0.0002142

Notes: CHR—chromosome; A1—minor allele (allele associated with a better therapeutic response); A2—major allele (allele associated with a poorer therapeutic response); OR—odds ratio with 95% confidence interval (CI); PASI—Psoriasis Area and Severity Index. The “/” symbol indicates that the rsID is located between two genes. CSP FMBA database—a national population frequency database of genetic variants in the Russian Federation based on more than 120,000 whole genomes.

**Table 2:** List of 12 genetic markers with the lowest *p*-values associated with psoriasis using an additive logistic regression model. Number of patients: 1026.

rsID	Gene	CHR	Genomic Position (hg19)	Effect Allele (A1)	Other Allele (A2)	Allele Frequency (A1)	OR (95% CI)	*p*
–	*ENSG00000302591/ENSG00000253619*	8	120,877,408	C	CTTTTTTTTTT	0.220	1.336 [1.173–1.521]	0.0000122
–	*LINC02355*	4	149,157,085	T	C	0.301	1.304 [1.158–1.469]	0.0000122
–	*ENSG00000253619*	8	120,933,873	C	T	0.259	1.317 [1.161–1.495]	0.0000195
–	*ZC3H8*	2	112,242,186	T	C	0.314	1.289 [1.145–1.451]	0.0000268
Haplotype 1	*ENSG00000253619*	8	–			0.239	1.315 [1.155–1.496]	0.0000329
–	*ENSG00000253619*	8	120,934,000	T	G	0.239	1.315 [1.155–1.496]	0.0000329
Haplotype 2	*ENSG00000253619*	8	–			0.260	1.307 [1.152–1.483]	0.0000334
rs1812780697	*ENSG00000253619*	8	120,933,689	A	G	0.260	1.307 [1.151–1.483]	0.0000339
Haplotype 3	*ENSG00000302591/ENSG00000253619*	8	–			0.186	1.339 [1.166–1.537]	0.0000345
Haplotype 4	*ENSG00000302591/ENSG00000253619*	8	–			0.186	1.339 [1.166–1.537]	0.0000346
–	*ENSG00000302591/ENSG00000253619*	8	120,879,772	C	CATTT	0.186	1.339 [1.166–1.537]	0.0000348
–	*ENSG00000302591/ENSG00000253619*	8	120,878,319	T	G	0.186	1.340 [1.166–1.539]	0.0000349

Notes: CHR—chromosome; A1—minor allele; A2—major allele; OR—odds ratio with 95% confidence interval (CI). The “/” symbol indicates that the rsID is located between two genes. Haplotype 1: chr8:120934004:G:T–chr8:120934008:G:T–chr8:120934012:G:T. Haplotype 2: chr8:120931307:T:C–chr8:120931611:A:G–chr8:120931825:C:G–chr8:120932612:T:C–chr8:120932805:G:C–chr8:120933341:A:G–chr8:120933463:A:C. Haplotype 3: chr8:120879600:G:A–chr8:120879843:G:A–chr8:120879872:T:C–chr8:120880386:G:T. Haplotype 4: chr8:120878187:G:A–chr8:120878202:A:G–chr8:120878223:A:G.

**Table 3:** List of 15 genetic markers with the lowest *p*-values associated with psoriatic arthritis using an additive logistic regression model. Number of patients: 96.

rsID	Gene	CHR	Genomic Position (hg19)	Effect Allele (A1)	Other Allele (A2)	Allele Frequency (A1)	OR (95% CI)	*p*
Haplotype 1	*LINC02363/* *LINC02362*	4	–			0.261	27.643 [5.690–134.286]	0.0000385
rs10758659	*RCL1*	9	4,858,019	G	A	0.484	0.057 [0.014–0.223]	0.0000410
Haplotype 2	*LINC02363/* *LINC02362*	4	–			0.250	25.883 [5.426–123.459]	0.0000447
Haplotype 3	*RCL1*	9	–			0.495	0.069 [0.019–0.250]	0.0000454
rs4861548	*FBLIM1/* *ENSG00000301674*	4	184,355,236	T	C	0.250	16.535 [4.279–63.888]	0.0000474
Haplotype 4	*ENSG00000307024*	11	–			0.293	6.806 [2.665–17.379]	0.0000609
rs13118751	*FBLIM1/* *ENSG00000301674*	4	184,354,335	T	C	0.261	16.026 [4.094–62.742]	0.0000678
rs4862186	*FBLIM1/* *ENSG00000301674*	4	184,355,256	T	C	0.245	15.262 [3.971–58.653]	0.0000726
Haplotype 5	*ENSG00000307024*	11	–			0.332	7.378 [2.735–19.897]	0.0000788
Haplotype 6	*RCL1*	9	–			0.500	0.102 [0.033–0.317]	0.0000797
Haplotype 7	*ENSG00000307024*	11	–			0.304	7.488 [2.753–20.363]	0.0000800
rs111996649	*ENSG00000307024*	11	127,444,368	A	AT	0.370	8.037 [2.849–22.672]	0.0000819
rs11279861	*TMEM132C*	12	128,552,486	GAAATTTTAT	G	0.141	25.800 [5.116–130.109]	0.0000824
rs35780244	*RCL1*	9	4,858,749	G	GA	0.500	0.078 [0.022–0.277]	0.0000825
Haplotype 8	*ENSG00000307024*	11	–			0.299	6.500 [2.558–16.518]	0.0000837

Notes: CHR—chromosome; A1—minor allele; A2—major allele; OR—odds ratio with a 95% confidence interval (CI). The symbol “/” indicates that the rsID is located between two genes. Haplotype 1—rs4990777-A/rs4990776-T/rs4997349-C. Haplotype 2—rs4862188-C/rs4862191-A/rs4862192-G/rs6842086-C/rs6819955-T. Haplotype 3—rs1576065-T/rs10815097-G. Haplotype 4—rs2078163-G/rs3853378-A/rs11220984-G/rs11220985-A/rs9787953-T. Haplotype 5—rs11220979-C/rs6590270-C. Haplotype 6—rs508227-T/rs1887430-A/rs10974817-G. Haplotype 7—rs356256-T/rs356253-T. Haplotype 8—rs4937251-A/rs7927195-A.

**Table 4:** Clinical characteristics of patients receiving TNF-α inhibitor therapy with good and insufficient treatment response.

Parameter	Good Response PASI ≥ 75 (*n* = 32)	Insufficient Response PASI ≤ 50 (*n* = 16)
Age, median (IQR), years	44.5 (36.5–56)	46.5 (38–58.5)
Sex (male/female), *n*	28/4	12/4
Disease duration, median (IQR), years	19.5 (13.5–30.5)	16.5 (10–26.5)
PASI, median (IQR)	25 (16.9–30.9)	19.8 (12.9–24.3)
BSA, median (IQR), %	25.2 (15.7–39)	23 (15.3–33)
Psoriatic arthritis, *n*	9	5
BMI, median (IQR), kg/m^2^	25.7 (23.5–29)	28.5 (26.5–34.3)
Normal body weight, *n*	13	2
Overweight (pre-obesity), *n*	14	6
Obesity class I, *n*	3	3
Obesity class II, *n*	4	1
Underweight, *n*	1	1
Arterial hypertension, *n*	7	9
Family history of psoriasis, *n*	7	8
Smoking, *n*	10	2
Alcohol abuse, *n*	1	0
Prior biologic therapy, *n*	5	4

## Data Availability

The genomic data are generated from patient samples and therefore are only available under restricted access.

## Supplementary Materials

The following supporting information can be downloaded at: https://www.mdpi.com/article/10.3390/ijms27104422/s1.

## Abbreviations

The following abbreviations are used in this manuscript:

AUC	Area Under the Curve
BMI	Body mass index
BSA	Body surface area
DLQI	Dermatology Life Quality Index
FDR	False discovery rate
GWAS	Genome-wide association study
IL	Interleukin
IQR	Interquartile range
MHC	Major histocompatibility complex
NMD	Nonsense-mediated decay
PASI	Psoriasis Area and Severity Index
PRS	Polygenic risk score
PsA	Psoriatic arthritis
SNP	Single-nucleotide polymorphism
TRM	Tissue-resident memory [T cells]
TNF-α	Tumor necrosis factor-alpha
